# Genotyping and plant-derived glycan utilization analysis of *Bifidobacterium* strains from mother-infant pairs

**DOI:** 10.1186/s12866-020-01962-w

**Published:** 2020-09-10

**Authors:** Zeyu Kan, Baolong Luo, Jingjing Cai, Yan Zhang, Fengwei Tian, Yongqing Ni

**Affiliations:** 1 School of Food Science and Technology, Shihezi University, Xinjiang, China; 2 School of Food Science and Technology, Jiangnan University, Wuxi, Jiangsu China

**Keywords:** *Bifidobacterium*, Glycan utilization, Genotype comparison, Fingerprint

## Abstract

**Background:**

Bifidobacteria are important probiotics; some of the beneficial effects of bifidobacteria are achieved by the hydrolysis of glycans in the human gut. However, because the diet of breastfed infants typically lacks plant-derived glycans, in the gut environment of mothers and their breastfed infants, the mother will intake a variety of plant-derived glycans, such as from onions and bananas, through her diet. Under this assumption, we are interested in whether the same species of bifidobacteria isolated from mother-infant pairs present a distinction in their hydrolysis of plant-derived carbohydrates.

**Results:**

Among the 36 *Bifidobacterium* strains, bifidobacterial carbohydrate utilization showed two trends related to the intestinal environment where the bacteria lived. Compared with infant-type bifidobacterial strains, adult-type bifidobacterial strains preferred to use plant-derived glycans. Of these strains, 10 isolates, 2 *Bifidobacterium pseudocatenulatum* (*B. pseudocatenulatum*), 2 *Bifidobacterium pseudolongum* (*B. pseudolongum*), 2 *Bifidobacterium bifidum* (*B. bifidum*), 2 *Bifidobacterium breve* (*B. breve*), and 2 *Bifidobacterium longum* (*B. longum*), were shared between the mother-infant pairs. Moreover, the repetitive sequence-based polymerase chain reaction (rep-PCR) results illustrated that *B. pseudolongum* and *B. bifidum* showed genotypic similarities of 95.3 and 98.2%, respectively. Combined with the carbohydrate fermentation study, these results indicated that the adult-type strains have a stronger ability to use plant-derived glycans than infant-type strains. Our work suggests that bifidobacterial carbohydrate metabolism differences resulted in the selective adaptation to the distinct intestinal environment of an adult or breastfed infant.

**Conclusions:**

The present study revealed that the different gut environments can lead to the differences in the polysaccharide utilization in the same strains of bifidobacterial strains, suggesting a further goal of investigating the exact expression of certain enzymes in response to specific carbon sources.

## Background

Human intestinal microorganisms constitute an additional organ that plays an irreplaceable role in maintaining human health and normal biological activity [1]. Bifidobacteria are among the most studied human intestinal bacteria and are indispensable microorganisms in the host gut [2]. Interestingly, studies have shown that bifidobacteria may be transmitted vertically from mother to child. Because *B. longum* subsp. *infantis* has the ability to utilize particular oligosaccharides in breast milk, it is believed to be colonized in the gut of newborns [1, 3]. In addition, microorganisms are randomly colonized in the initial intestinal tract, and the colonized species will be affected by the intestinal environment [4, 5]. Non-digestible glycan is considered to be a critical energy sources and is thought to be responsible for the survival and proliferation of many microorganisms in the gut microbiome; thus, dietary intake of glycan is an important environmental factor for intestinal microorganisms [6–8]. The predominant diet of breastfed infants is human milk, which lacks plant-derived glycans [9]. However, adults can acquire most of glycans [10, 11]^,^ which include plant-derived sources of xylo-oligosaccharide (XOS), raffinose, stachyose, resistant starch (RS), inulin, fructo-oligosaccharide (FOS) and isomalto-oligosaccharide (IMO) [6, 12, 13], and the other sources of galacto-oligosaccharide (GOS). Meanwhile, FOS and GOS are believed to increase some bacterial populations, such as those of bifidobacteria [13–15]. However, only a few species of bifidobacteria can utilize XOS and inulin such as *B. animalis* ssp. *lactis* [13, 16]. Because breastfed infants and their mothers ingest completely different glycans [9, 11], it is worth investigating whether the same species of bifidobacteria isolated from mother-infant pairs present similarities in the utilization of plant carbohydrates. However, some studies have only assessed which species of oligosaccharides are utilized by bifidobacteria [15], and little is known about the carbohydrate metabolism of bifidobacteria in relation to the mother-infant pairs.

Based on genomics studies, genes related to carbohydrate metabolism accounted for only 12.4% of bifidobacterial open-reading frame genes [17], which explains why different species of bifidobacteria exert the ability to use different plant-derived carbohydrates [18]. Interestingly, glycosyl hydrolases (GHs), which are members of the GH3 and GH43 families, are recognized to have a universal relationship with the degradation of plant polysaccharides [1, 19], which may explain why bifidobacteria might be a key strain among the intestinal microflora in humans or other animal species [18]. Recently, researchers have gradually discovered many metabolic mechanisms by which bifidobacteria breakdown polysaccharides, including unique metabolic pathways (the bifid-shunt) and the expression of GH family members [1, 20].

Using the repetitive sequence-based polymerase chain reaction (rep-PCR) technique, bifidobacterial genotyping can be achieved, and genotype analyses can be employed to distinguish species and stains [21, 22]. Extended analysis of genotypes can also be applied to analysis of bifidobacteria from mother-infant pairs and is a preliminary screening approach and a study on the genetic correlation [22].

However, it is not clear what differences in bifidobacteria glycan metabolism occur between mothers and infants as a result of vastly different carbohydrate intakes in the gastrointestinal tract between mothers and breastfed infants.

In this study, we used *Bifidobacterium* isolates (*Bifidobacterium longum* subsp. *longum* or *Bifidobacterium longum*, *Bifidobacterium breve, Bifidobacterium pseudocatenulatum,* and *Bifidobacterium bifidum*) that were previously found in fecal samples of both adults and infants [23]. We evaluated the ability of plant-derived carbohydrates to promote the growth of different species of bifidobacteria. The aim of this paper was to compare plant-derived carbohydrate fermentation results and genotyping results using rep-PCR fingerprints of *Bifidobacterium* species isolated from the feces of mothers and their paired infants.

## Results

### The use of bifidobacterial strains

Bifidobacteria isolates were isolated from 20 pairs of mother-infant fecal samples; in total, there were 36 bifidobacterial isolates (Table 1); *B. pseudocatenulatum* (*n* = 12), *B. pseudolongum* (*n* = 9), *B. bifidum* (*n* = 7), *B. breve* (*n* = 4), *B. longum* (*n* = 4). Among these bifidobacterial isolates, we obtained 5 pairs of isolates. These 5 pairs of isolates were present in mother feces (*n* = 5) and infant feces (*n* = 5): 2 *B. pseudocatenulatum*, 2 *B. longum*, 2 *B. breve*, 2 *B. bifidum*, and 2 *B. pseudolongum*. In subsequent trials, we will plan to use genome sequencing and comparative genomics to further study the 5 pairs of bifidobacterial isolates. The data have been deposited in the sequence read archive (SRA) of the NCBI as GenBank Accession Number MT826639-MT826674.

**Table 1:** The number of bifidobacterial strains used in this study

Sample	*B. pseudocatenulatum*	*B.pseudolongum*	*B.bifidum*	*B. breve*	*B. longum*	Total
Infant fece	4	5	6	2	3	20
Adult fece	8	4	1	2	1	16

*B. pseudocatenulatum*, *Bifidobacterium pseudocatenulatum*; *B. pseudolongum*, *Bifidobacterium pseudolongum*; *B. bifidum*, *Bifidobacterium bifidum*; *B. breve*, *Bifidobacterium breve*; *B. longum*, *Bifidobacterium longum*

### Analysis of carbohydrate utilization by bifidobacteria

**Fig. 1:**
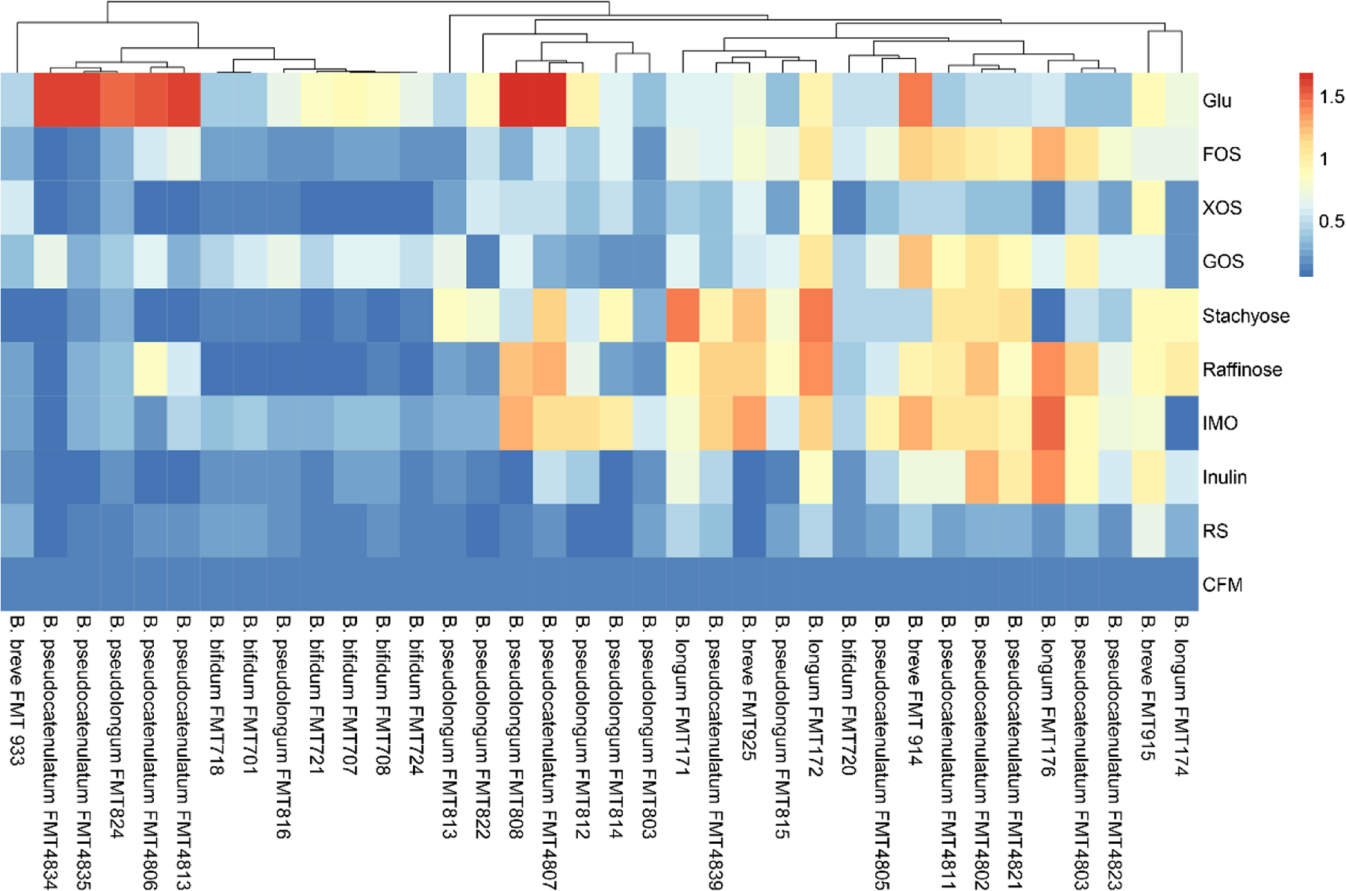
Carbohydrate utilization data of 36 strains of bifidobacteria were visualized by heatmap. FMT, Food Microbiology Technology Laboratory

The carbohydrate consumption data for the 36 bifidobacterial strains and 9 different carbon sources are shown in Fig. 1. The final growth profiles were divided into four growth ability groups: no growth (final OD_600 nm_ < 0.3), limited growth (final OD_600 nm_ = 0.3–0.5), moderate growth (final OD_600 nm_ = 0.5–0.8), and good growth (final OD_600 nm_ > 0.8). In carbon-free media without any carbohydrate supplementation, no bifidobacterial strains displayed significant growth. This result confirms that the CFM is an are appropriate medium to evaluate the carbohydrate-metabolizing ability of the majority of the bifidobacterial strains tested in this study.

All of the bifidobacterial strains tested in the positive control (with Glu as the substrate) displayed obvious growth after 48 h of cultivation. Apart from Glu, twenty-one or twenty-two of the thirty-six bifidobacterial strains reached moderate growth or higher when FOS or GOS was used as a single carbon source, respectively. XOS supported vigorous growth of bifidobacteria in only nine of the isolates utilized in test. When XOS was the carbon source, only two strains, *B. breve* FMT 915 and *B. longum* FMT172, showed good growth, which final OD_600 nm_ values of 0.880 ± 0.028 and 0.852 ± 0.012 (Additional file 1), respectively. In addition, when IMO was the carbon source, 19 strains of bifidobacterial exhibited OD_600 nm_ of more than 0.5; when stachyose was the carbon source, the number of strains was 18; and when raffinose was the carbon source, the number of strains was 20. Long-chain fructose polymers, such as inulin, stimulated the growth of 15 bifidobacterial strains, and notably, all *B. longum* strains could utilize inulin when it was the sole carbon source tested. Moreover, only ten bifidobacterial strains could grow in RS, with just one strain, *B. breve* strain FMT915, reached a final OD_600 nm_ of 0.666 ± 0.037; while the growth of other strains was limited.

### Analysis of bifidobacteria interspecies differences in terms of carbohydrate metabolism

**Fig. 2:**
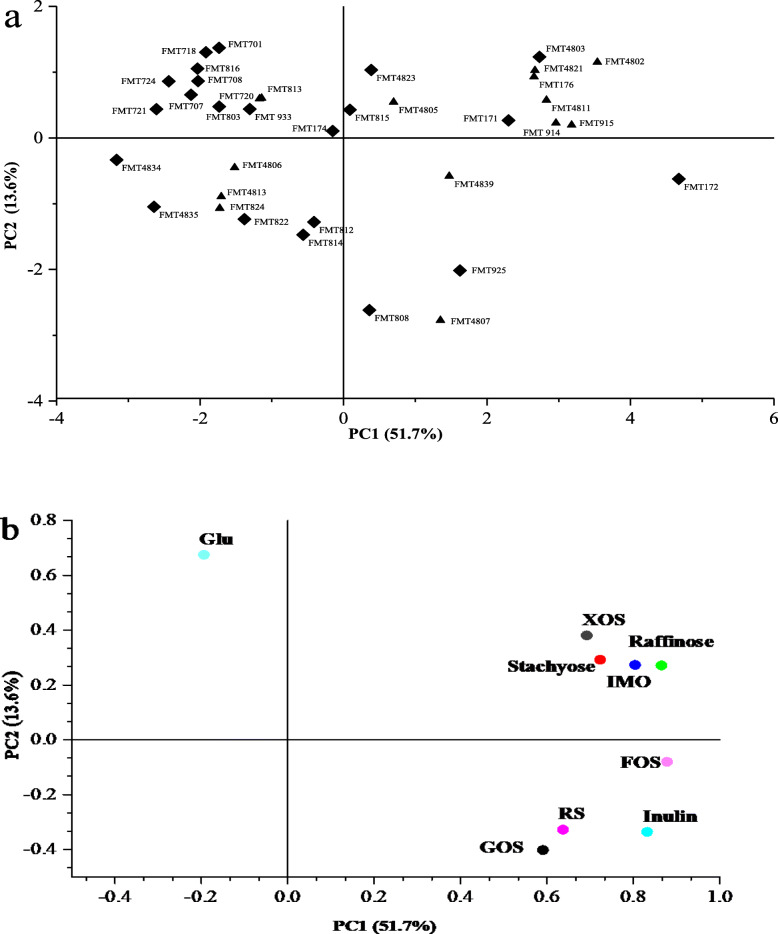
Principal component analysis (PCA) defined by the two principal components (PC1 and PC2) of the maximum OD_600 nm_ values of all bifidobacteria strains growth on GOS, FOS, XOS, IMO, RS, Glu, Inulin, Raffinose, Stachyose. (**a**) represents bifidobacterial strains (rhombus represents infant-type bifidobacteria, the triangle represents adult-type bifidobacteria) and (**b**) represents carbon sources. GOS, galacto-oligosaccharide; FOS, fructo-oligosaccharide; XOS, xylo-oligosaccharide; IMO, isomalto-oligosaccharide; RS, resistant starch; Glu, glucose; FMT, Food Microbiology Technology Laboratory

Using PCA, we evaluated the maximum OD_600 nm_ values of each strain grown on every carbon source tested in this study (Fig. 2). Two PCs accounted for 65.3% of the total variance (PC1 51.7%; PC2 13.6%). PC2 was defined by Glu growth, which was positively correlated with most of the test strains and differed when compared with the other carbon sources. The descriptors IMO, stachyose, RS, FOS, raffinose, inulin, and GOS were principally explained by PC1. The clustering of these variables showed that bifidobacterial strains grew on stachyose similarly to IMO and raffinose. Meanwhile, the maximum OD_600 nm_ values on GOS and RS were similar to each other (Fig. 2b). Moreover, *B. bifidum* strains clustered more tightly together than the other four species of bifidobacterial strains. Interestingly, with respect to the source of the strains, half of the bifidobacterial strains from adult feces were clustered together and strongly consumed the plant-derived glycans. In contrast, with respect to strains from infant sources, more than half of the bifidobacterial strains clustered together and consumed small amounts of the plant-derived glycans (Fig. 2a). Collectively, our results showed that the growth phenotype in response to plant-derived carbohydrates is mostly maintained by adult-type bifidobacterial strains. Most of the species of the infant-type bifidobacterial strains lack the ability to consume plant-derived carbohydrates.

### Comparison of the carbohydrate fermentation and genotypes of bifidobacteria from mother-infant pairs

**Fig. 3:**
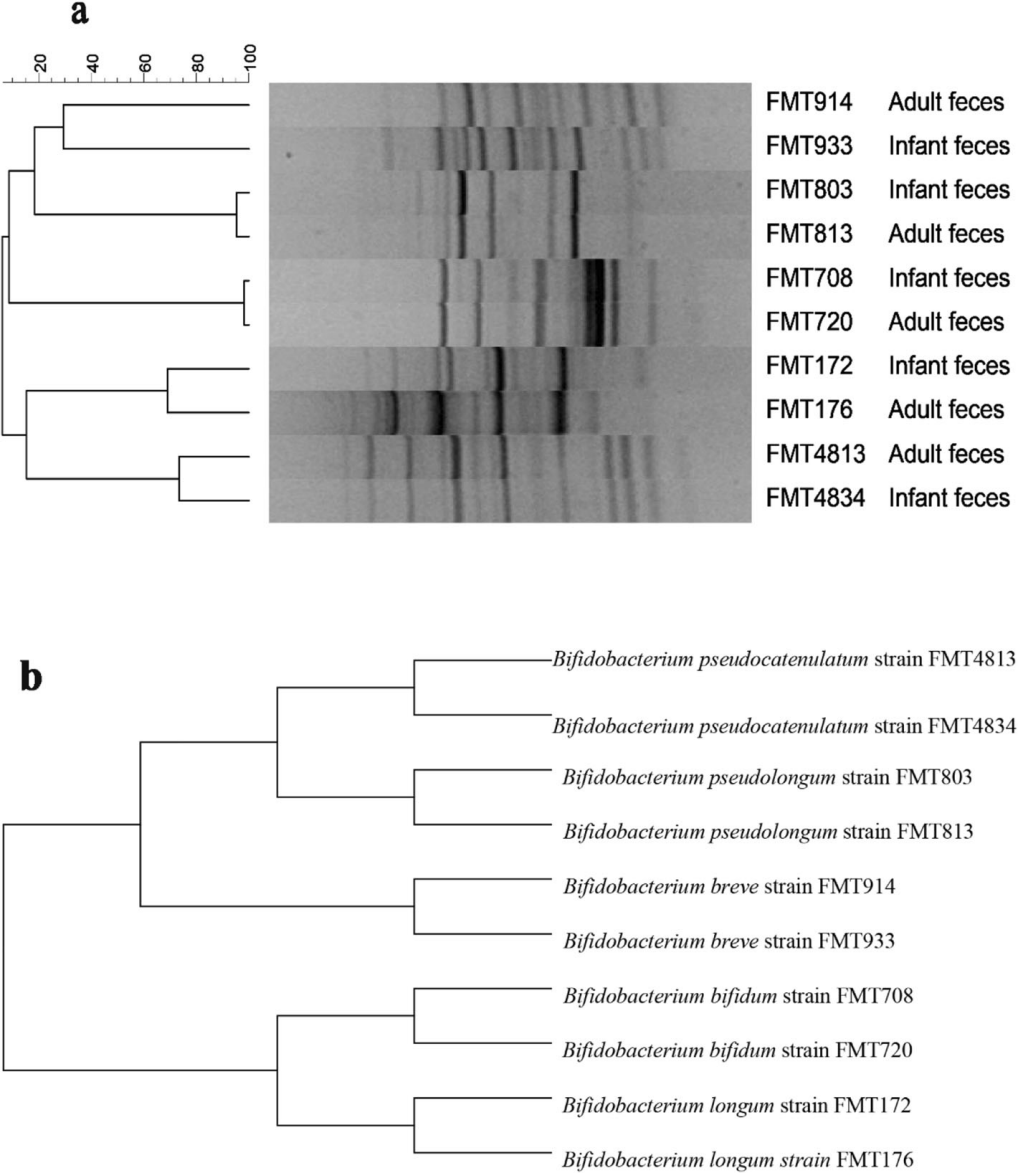
Dendrogram based on (**a**) the Rep-PCR fingerprints of genomic and (**b**) 16S rRNA sequence evolutionary tree from the 10 strains of mother infant paired bifidobacteria. *B. pseudocatenulatum* (FMT4834 and FMT4813), *B. breve* (FMT914 and FMT933), *B. pseudolongum* (FMT803 and FMT813), *B. bifidum* (FMT720 and FMT708) and *B. longum* (FMT172 and FMT176). Rep-PCR, Repetitive sequence-based polymerase chain reaction; FMT, Food Microbiology Technology Laboratory

**Fig. 4:**
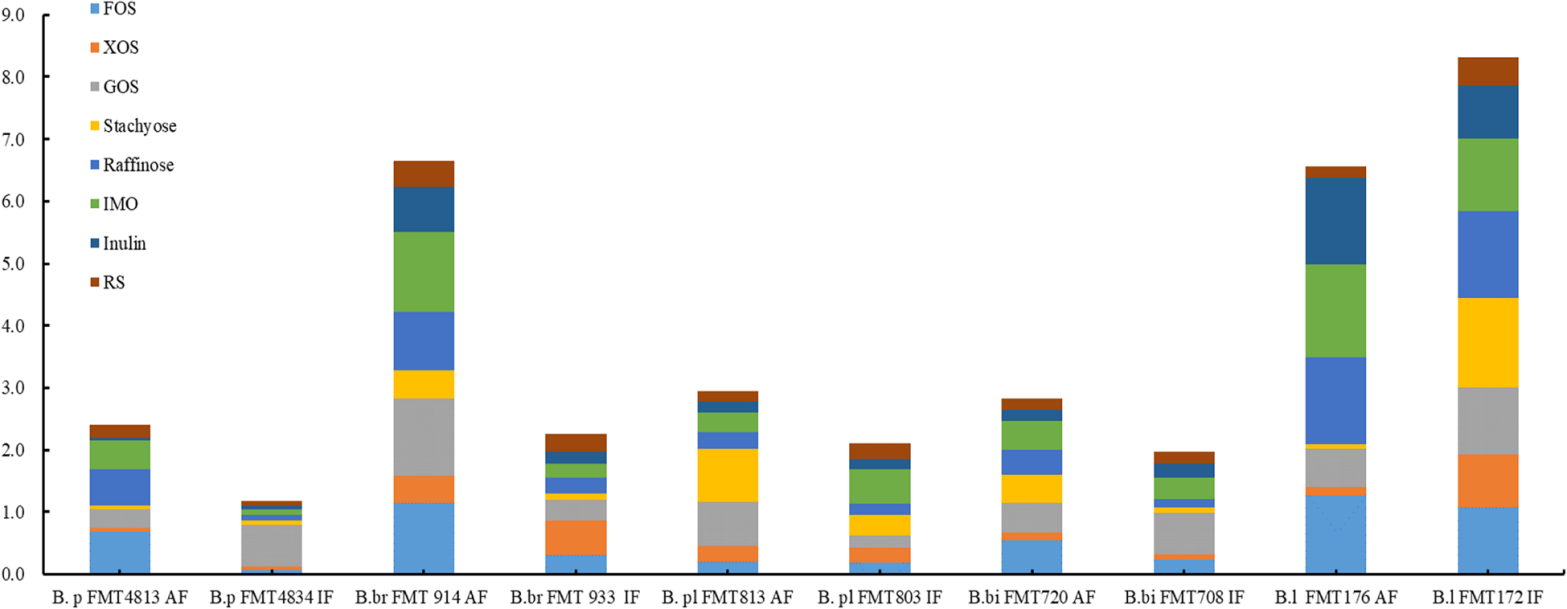
The final OD_600 nm_ values of ten mother-infant paired bifidobacterial strains for 48 h in eight plant-derived glycans. B.p FMT4834 and FMT4813, B.br FMT914 and FMT933, B.pl FMT803 and FMT813, B.bi FMT720 and FMT708, B.l FMT172 and FMT176. B.p, *B. pseudocatenulatum*; B.br, *B. breve*; B.pl, *B. pseudolongum*; B.bi, *B. bifidum*; B.l, *B. longum*; AF, adult feces; IF, infant feces; FMT, Food Microbiology Technology Laboratory

We compared the carbohydrate fermentation data (Fig. 4). For *B. pseudocatenulatum*, the ability of mother-derived FMT4813 ability to utilize FOS, GOS, stachyose and raffinose resulted in limited growth or more; moreover, infant-derived FMT4834 could consume only GOS. Regarding the *B. breve* (FMT914 and FMT933) from mother-infant pairs, mother-derived FMT914 was capable of utilizing all tested carbon sources and reached a final OD_600 nm_ on FOS, GOS and IMO of more than 1.0 (1.144 ± 0.038, 1.241 ± 0.057 and 1.288 ± 0.131, respectively), while the final OD_600 nm_ values in response to XOS, stachyose, raffinose, inulin and RS were 0.436 ± 0.013, 0.450 ± 0.030, 0.945 ± 0.060, 0.731 ± 0.060 and 0.408 ± 0.006, respectively (Additional file 1). However, the infant-derived FMT933 strains displayed growth only on FOS, XOS and GOS alone, with final OD_600 nm_ values between 0.3 and 0.6 (Fig. 4). By analyzing the glycan fermentation data, we found that the glycan utilization of bifidobacterial strains from mother-infant pairs showed differences. In experimental data (Fig. 4), with the exception of *B. longum*, in the 4 other groups of mother-infant pairs, the mother-derived bifidobacterial strains showed a stronger ability to utilize glycans than the infant-derived strains. Although they are relatively closely related from the point of view of the evolutionary tree (Fig. 3b), compared with the bifidobacteria that were isolated from the feces from mothers, those from the feces of exclusively breastfed infants were less able to use plant-derived glycans. In addition, both *B. pseudolongum* (mother-derived FMT813 and infant-derived FMT803 showed a 95.3% genotype similarity) and *B. bifidum* (mother-derived FMT720 and infant-derived FMT708 showed a 98.2% genotype similarity) showed similar results (Fig. 3a and Fig. 4). These results indicate that the mother-derived bifidobacteria utilized a wide spectrum of plant-driven carbohydrates.

## Discussion

The ability of bifidobacteria to ferment complex carbohydrates plays an important role in their own colonization of the colon and has the potential to sustain other microorganisms [24]. Our work showed that only a few bifidobacterial strains among the 36 tested strains could consume inulin or RS in pure culture. Consistent with a previous study said, *B. pseudocatenulatum* strains were the most capable to utilize inulin and grow [12, 18]. In addition, a previous study found that *B. breve* UCC2003 is able to utilize the starch because it may encode the required associated hydrolases [25]. Interestingly, our results also found that *B. breve* FMT915 possessed good RS utilization ability (final OD_600 nm_ of 0.666 ± 0.037), and this is a point worth studying in depth (Fig. 1). Research on interspecies metabolic differences has shown that bifidobacterial strains can effectively use carbohydrates via specificity within species of bifidobacteria [2]. Our results also support this the conclusion. Furthermore, compared with the other bifidobacterial strains in this study, *B. breve* FMT915 had a stronger ability to utilize the various carbohydrates in the presence of Glu, FOS, XOS and GOS, which reached a final OD_600 nm_ values of 0.47 ± 0.033, 0.313 ± 0.031, 0.553 ± 0.026, and 0.329 ± 0.027, respectively (Additional file 1). On the other hand, carbohydrate utilization profiles have been previously demonstrated to be differ within species [12].

According to the data (Fig. 1), the same species of bifidobacterial strains can differ in their ability to utilize carbon sources. Seven *B. bifidum* strains were able to grow in the Glu; however, expect for *B. bifidum* FMT720, most *B. bifidum* could not grow when FOS, stachyose or raffinose was the sole carbon source, which showed the final OD_600 nm_ values of 0.548 ± 0.040, 0.442 ± 0.03 and 0.401 ± 0.007, respectively (Additional file 1). The test results showed that *B. bifidum* utilized the plant-derived glycan poorly, and this reflects the fact that *B. bifidum* belongs to the mucosa glycan utilization- associated bifidobacterial communities [2]. A previous study found that only *Bifidobacterium lactis* strains have preferences amongst XOS [16]; however, we found that *B. longum* FMT172 grew well (final OD_600 nm_ of 0.852 ± 0.012). *Bifidobacterium lactis* and *B. adolescentis* were not the only isolates that could metabolize XOS to a large extent [16], some isolates of *B. longum* also metabolized XOS [26]. The four strains of *B. longum* achieved moderate growth or more on inulin, and it has been reported that β-fructofuranosidase enzymes can show specificity for inulin [13, 27]. This specificity may explain why *B. longum* has a strong inulin utilization ability for inulin, which was also shown by SHIME experiments [28].

### Comparison of the genotypes of bifidobacteria from mother-infant pairs

In genotype clustering analysis, isolates from the feces of mothers and their infants displayed similar genotypes; UPGMA clustering of (GTG)_5_-PCR at the species level revealed ranges of 29.3 to 98.2% similarity. In particular, the *B. pseudolongum* and *B. bifidum* from mother-infant pairs showed 95.3 and 98.2% genotypic similarity (Fig. 3a), respectively. Several studies have identified genomically identical bifidobacterial strains from stool samples of mothers and their infants [1, 5]^.^ However, previous studies have indicated that the species and strains of *Bifidobacterium* have single utilization or interspecific metabolic cooperation in use of plant-derived glycan [1, 2, 28]. By constructing a phylogenetic tree, we found that these bifidobacterial strains are closely related (Fig. 3b). Through bifidobacterial phage experiments, one study demonstrated that bifidobacteria spread vertically from mother to child [29]. Bifidobacteria need to adjust gene expression quickly because of changing intestinal environmental conditions [30]. In these five pairs of mother to infant bifidobacteria, we wanted to explore the transfer relationship of bifidobacteria from mother to baby from the perspective of plant-derived polysaccharide hydrolysis.

Although genotypically similar bifidobacterial strains were isolated from mothers and their paired infants, the carbohydrate consumption profiles in this study revealed that the adult-type bifidobacteria were capable of utilizing a greater variety of glycans than the infant-type isolates. The intestinal environment may potentially be related to regulation of bifidobacterial-related gene expression [30]. Over time, the adult microflora reached a stable dynamic community, and a unique population was found in each individual [31]. Bifidobacteria are already suited to utilizing complex plant-derived glycans, while breastfed infant bifidobacteria are universally weak in terms of their consumption ability because the suckling period of infants is associated with a lack of plant-derived carbohydrates.

## Conclusions

In summary, our data show that bifidobacterial carbohydrate metabolism differences resulted in the selective adaptation to the distinct intestinal environment of the adult or breastfed infant. To clarify plant-derived glycan metabolism differences between similar bifidobacteria, we need to further investigate the exact expression of some enzymes in response to specific carbon sources; in particular, *B. breve* can utilize XOS as the sole carbon source.

## Methods

### Fecal samples and Bifidobacteria isolation method

Twenty paired fecal samples were obtained from mothers and their infants were obtained from the remote rural area of Kashgar, Xinjiang, China. Notably, in this study, infants (less than 8 months of age) were exclusively breastfed. Writing testimonial and ethical approval were obtained from the infant’s parents and Research Ethics Committee of Shihezi University. The number of the ethical approval JN.No20190315c0400418.

The fecal samples were homogenized and then serially diluted with sterilized normal saline solution containing 0.05% (w/v) L-cysteine (Blotopped, China). Appropriate samples dilutions prepared and spread on a Man-Rogosa-Sharpe (MRS) solid-medium plates supplemented with 0.05% (w/v) L-cysteine, 50 mg/L mupirocin (Bomei, Hefei, China) and 25 mg/L nystatin (Bomei, Hefei, China) [32]. The solid plates were incubated in a DG250 anaerobic workstation (Don Whitley Scientific, UK) at 37 °C for 48 h. The colonies that showed different morphological features were isolated for subsequent analysis via 16S rRNA gene sequencing of genomic DNA.

### Bacterial strains and media

All bifidobacterial strains in this study used were provided by the Laboratory of Food Microbiology Technology (FMT), School of Food Science and Technology, Shihezi University. Thirty-six bifidobacterial strains belonging to five species were used in this study (Table 1). The strains were anaerobically cultured at 37 °C for 48 h in MRS media [33] supplemented with 0.05% (w/v) L-cysteine. All the strains were stored in MRS broth supplemented with 50% (v/v) glycerol (Sinopharm Chemical Reagent Beijing Co., Ltd., China) and stored at − 80 °C.

### Carbohydrates fermentation experiments

Bifidobacterial strains were cultivated anaerobically at 37 °C for 48 h on MRS agar media. After selecting a single colony, enrichment was cultured in fresh MRS media for 18 h, after which the cultivation tubes were centrifuged at 5000 g for 5 min, washed and resuspended in PBS. Cell suspensions (approximately 10^7^CFU/mL) were used to prepare the bacterial inoculants for the carbohydrate fermentation experiments.

The carbohydrate utilization of bifidobacteria was investigated in the carbohydrate-free media (CFM) as previously described by adding the individual carbohydrates [34]. The CFM comprised tryptone (5.0 g/L), yeast extract (5.0 g/L), peptone (10 g/L), K_2_HPO_4_ (2.0 g/L), diammonium citrate (2.0 g/L), sodium acetate (5.0 g/L), manganese sulfate monohydrate (0.25 g/L), magnesium sulfate heptahydrate (0.58 g/L), Tween 80 (1 ml/L), and L-cysteine HCl (0.5 g/L) (Bomei, Hefei, China). All the other reagents were purchased from Sinopharm Chemical Reagent Beijing Co., Ltd., China. The pH of the CFM was adjusted to a final pH of 6.8 after autoclaving at 121 °C for 20 min. The eight commercial carbohydrates were filter sterilized by 0.22 μm filters (Bomei, Hefei, China), after which different 1% (w/v) carbon solutions were added to the CFM. The commercial carbohydrates were purchased from Yuanye Biotechnology Co., Ltd., Shanghai, China, and included the following: FOS (purity ≥95%), GOS (purity ≥90%), XOS (purity ≥95%), IMO (purity ≥90%), raffinose (purity ≥98%), stachyose (purity ≥85%), inulin (purity ≥90%) and RS (from maize, types RS3) [35]. Moreover, glucose (Glu) was used as a positive control without the addition of carbon sources as a negative control. Then media were then inoculated with different 1% (v/v) bifidobacteria cultures. Each carbon source fermentation experiment was performed in an anaerobic environment at 37 °C for 48 h. Experiments were performed for each bifidobacterial strain and carbohydrate combination, with three independent biological replicates.

### Assessing the growth of bifidobacteria on different carbon sources

The growth of the bifidobacterial strains was monitored via a visible-wavelength spectrophotometer (Eon Microplate Spectrophotometer; Bio Tek, Winooski, VT, USA) to determine the final OD_600 nm_ values. In Fig. 1, Carbohydrate utilization data of 36 strains of bifidobacteria were visualized by heatmap [36] and an additional spreadsheet file shows this in more detail (see Additional file 1).

### Genotype fingerprinting analysis of bifidobacteria by rep-PCR

The isolation of genomic DNA was extracted by the CTAB method as described previously [37]. Repetitive sequence-based polymerase chain reaction (rep-PCR) used the (GTG)_5_ primer (5′-GTGGTGGTGGTGGTG-3′) [22]. After electrophoresis, Gelcompar II version 6.6 (Applied Maths, Sint-Matenslatem, Belgium) was used to analyze the images of the amplicon fingerprint. The software analyzed the rep-PCR profiles and made use of Pearson correlation analysis to read the banding patterns, and a dendrogram was created by the unweighted pair group method with arithmetic averages [28]. The raw data for the REP-PCR fingerprint was an additional figure file shows this in more detail (see Additional file 2).

### Statistical analysis

All the data for carbohydrate formation were principal components analysis (PCA) by SPSS 22.0 (SPSS Inc., Chicago, IL, USA). Evolutionary trees were analyzed by MEGA 7.0 software. The data was plotted by origin 9.0.1 software (Origin Lab, USA) and Excel 2016 (Microsoft, USA). Heatmap and dendrograms were constructed in R (ver 3.3.1) using the gplots package ver 3.0.1.

## Supplementary information

**Additional file 1.**

**Additional file 2.**

## Data Availability

The bifidobacteria of raw sequencing data are accessible through the accession number PRJNA650231, and the NCBI as GenBank Accession Number MT826639-MT826674 (this is the serial login number), and hyperlink to dataset in https://www.ncbi.nlm.nih.gov/nuccore/?term=MT826639:MT826674[accn].

## Abbreviations

FMT: Food microbiology technology laboratory
CFM: Carbon-free medium
MRS: Man-rogosa-sharpe medium
rep-PCR: Repetitive sequence-based polymerase chain reaction
