# Supplementary figures and images for: Genotyping and plant-derived glycan utilization analysis of Bifidobacterium strains from mother-infant pairs
Source: BMC Microbiol. 2020 Sep 10;20:277. doi: 10.1186/s12866-020-01962-w (PMC7488109; doi:10.1186/s12866-020-01962-w)

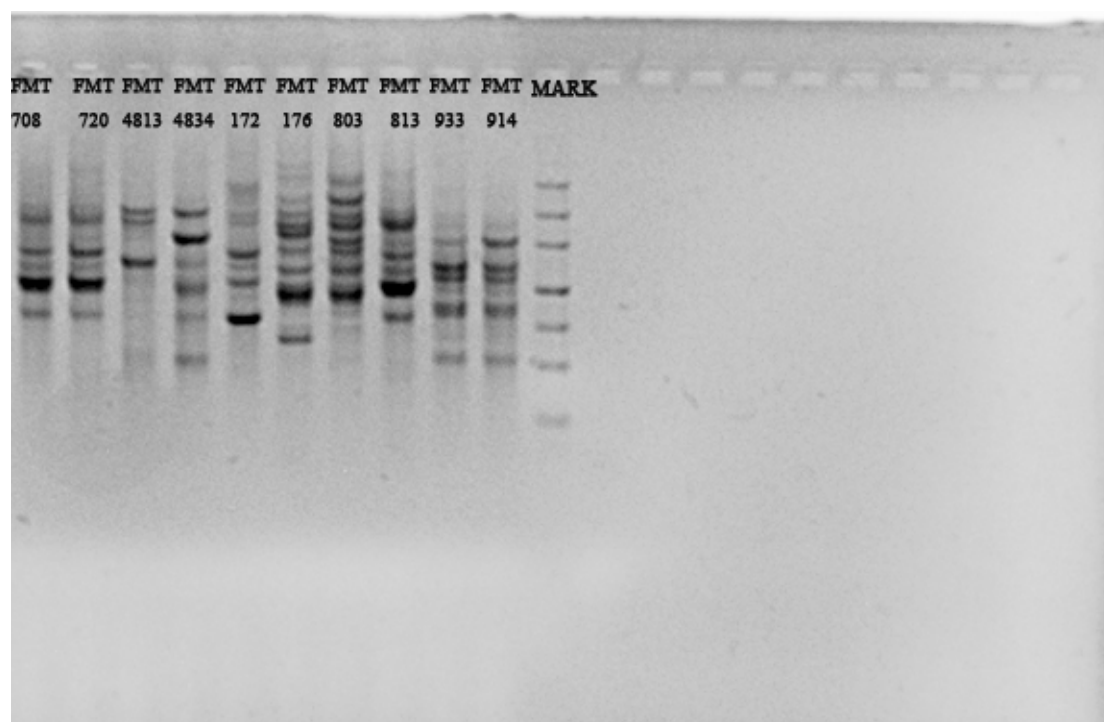

1

---

<sup>1</sup> FMT, Food Microbiology Technology Laboratory.

Supplement: Supplementary file 2 — Additional file 2. [file 12866_2020_1962_MOESM2_ESM.pdf]
